# HSPC300 and its role in neuronal connectivity

**DOI:** 10.1186/1749-8104-2-18

**Published:** 2007-09-25

**Authors:** Abrar Qurashi, H Bahar Sahin, Pilar Carrera, Alexis Gautreau, Annette Schenck, Angela Giangrande

**Affiliations:** 1 Institut de Génétique et de Biologie Moléculaire et Cellulaire, CNRS/INSERM/ULP, BP 10142, 67404 Illkirch, CU de Strasbourg, France; 2 Department of Human Genetics, Emory University School of Medicine, Atlanta, GA 30322, USA; 3 Abteilung für Molekulare Entwicklungsbiologie, Institut für Molekulare Physiologie und Entwicklungsbiologie, Universität Bonn, D-53115 Bonn, Germany; 4 Laboratoire de Morphogenèse et Signalisation Cellulaires, UMR 144 CNRS/Institut Curie, 75248 Paris Cedex 05, France; 5 Department of Human Genetics (855), Nijmegen Centre for Molecular Life Science, Radboud University Nijmegen Medical Centre, Box 9101, 6500 HB Nijmegen, The Netherlands

## Abstract

**Background:**

The WAVE/SCAR complex, consisting of CYFIP (PIR121 or Sra1), Kette (Nap1), Abi, SCAR (WAVE) and HSPC300, is known to regulate the actin nucleating Arp2/3 complex in a Rac1-dependent manner. While *in vitro *and *in vivo *studies have demonstrated that CYFIP, Kette, Abi and SCAR work as subunits of the complex, the role of the small protein HSPC300 remains unclear.

**Results:**

In the present study, we identify the *HSPC300 *gene and characterize its interaction with the WAVE/SCAR complex in the *Drosophila *animal model. On the basis of several lines of evidence, we demonstrate that HSPC300 is an indispensable component of the complex controlling axonal and neuromuscular junction (NMJ) growth. First, the *Drosophila HSPC300 *expression profile resembles that of other members of the WAVE/SCAR complex. Second, *HSPC300 *mutation, as well as mutations in the other complex subunits, results in identical axonal and NMJ growth defects. Third, like with other complex subunits, defects in NMJ architecture are rescued by presynaptic expression of the respective wild-type gene. Fourth, HSPC300 genetically interacts with another subunit of the WAVE/SCAR complex. Fifth, HSPC300 physically associates with CYFIP and SCAR.

**Conclusion:**

Present data provide the first evidence for HSPC300 playing a role in nervous system development and demonstrate *in vivo *that this small protein works in the context of the WAVE/SCAR complex.

## Background

The evolutionarily conserved WAVE/SCAR complex has emerged as an important Rac1 small GTPase downstream effector that regulates several aspects of neuronal architecture. The mammalian WAVE/SCAR complex is composed of five proteins: CYFIP (PIR121 or Sra1), Kette (Nap1 or Hem2), Abi (or Abl interactor), SCAR (WAVE) and HSPC300 [1,2]. Whereas in the mouse nervous system WAVE function has so far been analyzed exclusively [3,4], *Drosophila *mutants are available for all but one subunit, HSPC300 [5-9]. Although our understanding is still far from complete, studies of these mutants and their protein partners have already uncovered that the WAVE/SCAR complex acts as a crucial hub, integrating and regulating various signaling pathways. The SCAR protein, probably the best-studied subunit, is a direct activator of the Arp2/3 actin nucleating complex [2,10], which is required for the formation of a branched actin network [11]. The other complex subunits physically interact to form the WAVE/SCAR complex but also associate with distinct proteins and control specific pathways. CYFIP is a direct Rac1 effector and signals to the Fragile X mental retardation protein (FMRP) [7,12,13], a regulator of local protein translation that controls, among other targets, key players of the actin machinery [14-16]. CYFIP and Kette both associate with the SH2 SH3 adapter protein Nck/DOCK [17,18].

Despite these diverse protein partners, loss of function phenotypes for *SCAR*, *CYFIP *and *Kette *in the nervous system are remarkably similar, if not even identical [8,19]. These phenotypes include defects in axon growth, branching and pathfinding, as well as abnormal growth and morphology of neuromuscular junctions (NMJs), a fly model system for synaptic plasticity [6-9,19,20]. Since it was found that mutations in any one of the three fly proteins leads to instability of its partners [8], consistent with data in cellular systems or other organisms [21-25], these phenotypes are most likely the result of multiple corrupted pathways normally associated with the three proteins.

HSPC300 (haematopoietic stem/progenitor cell protein 300), a small protein of 8 kDa, is the most conserved subunit of the SCAR/WAVE complex and has recently come into focus for its essential role in plant cytoskeleton remodeling [26,27]. Mutations in *Brick1*, one of the *Arabidopsis HSPC300 *orthologs, cause morphological defects that are associated with loss of cortical F-actin enrichment and that are in agreement with a role for HSPC300 in promoting Arp2/3 complex activity [26-28]. While these data support a crucial role of HSPC300 in plant actin remodeling, others have shown that HSPC300 *in vitro *is neither required for assembly of the SCAR/WAVE complex [29] nor impacts on Arp2/3-dependent actin polymerization [22]. Also, RNA interference (RNAi)-mediated knockdown of HSPC300 in cultured *Drosophila *cells results in a reduction of cortical F-actin and alterations in cell morphology that are much weaker than those resulting from RNAi-mediated ablation of all other four subunits [23]. Altogether, these observations make it difficult to predict the importance of HSPC300 in the animal kingdom and its role as a subunit of the WAVE/SCAR complex. This is particularly relevant given the observation that vertebrate HSPC300 interacts with both SCAR and Abi, a subunit of the WAVE/SCAR complex that also interacts with and regulates WASP, another activator of Arp2/3 [29-31].

We here characterize HSPC300 in an animal model, with particular focus on nervous system development. Loss of HSPC300 recapitulates all aspects of nervous system defects characterizing SCAR, CYFIP and Kette mutants, but notably does not lead to abnormal cell fate choices in sensory organs, an Abi and WASP related function [5,9]. This result, together with genetic and biochemical interaction data, clearly demonstrates the importance of HSPC300 in the nervous system as well as its role in function and integrity of the WAVE/SCAR complex.

## Results

### HSPC300 accumulates in axons of the central nervous system

**Figure 1:**
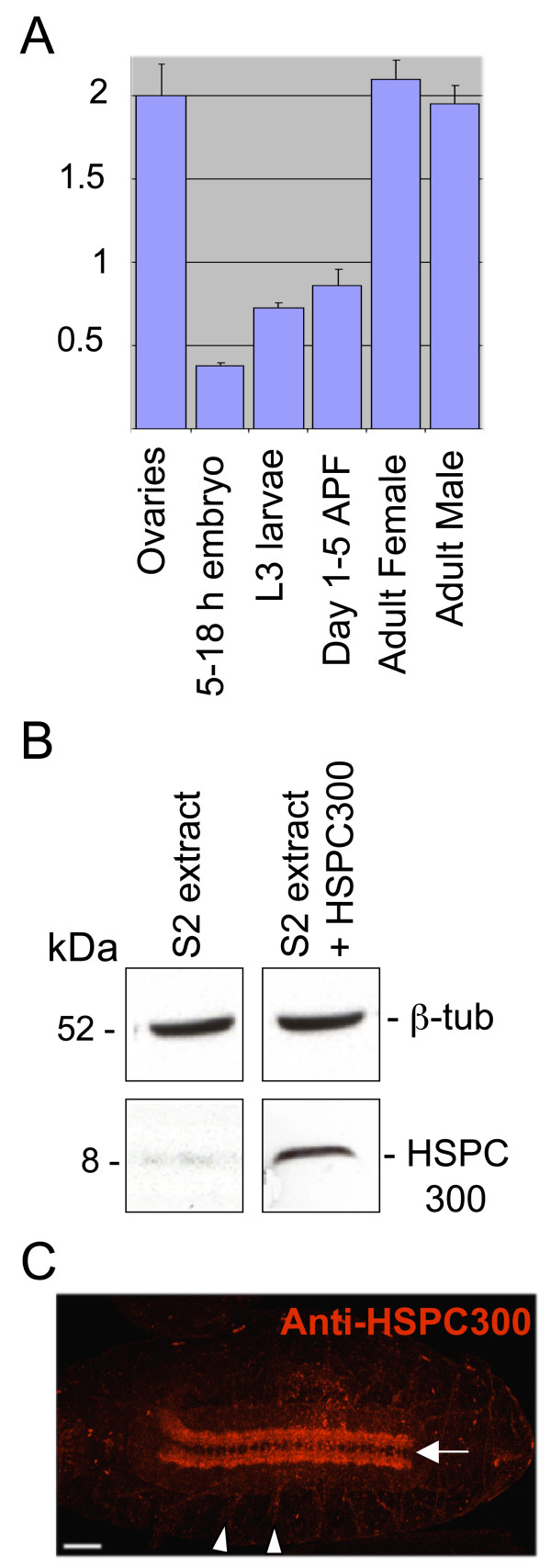
HSPC300 expression profile. **(a) **Quantitative analysis of *HSPC300 *mRNA levels by light cycler at indicated developmental stages. Quantification is relative to the housekeeping ribosomal protein 49 (rp49) mRNA. Bars indicate SEM. APF: After puparium formation. **(b) **Western blot analysis on protein extracts prepared from the *Drosophila *S2 cell line (S2 extract), using anti-HSPC300 antibody (HSPC300). The right lane represents equivalent amounts of protein extract from the S2 cell line, which transiently overexpresses HSPC300 upon transfection (S2 extract + HSPC300). β-tubulin (β-tub) represents a loading control. **(c) **HSPC300 immunolabeling of a whole mount embryo at stage 16; ventral view, anterior to the left. HSPC300 shows specific accumulation in CNS longitudinal connectives and commissures. The arrow and arrowheads show midline and motor neuron labeling, respectively. Scale bar: 50 μm.

A putative ortholog of the mammalian HSPC300 subunit of the WAVE/SCAR complex is annotated in FlyBase as *CG30173*. Using the available sequence information, we cloned *Drosophila HSPC300 *by RT-PCR and found it to be identical to the predicted gene sequence. In order to assess the expression profile of *HSPC300*, we performed quantitative real time RT-PCR on total RNA extracted from different developmental stages and determined that *HSPC300 *transcripts are present throughout the fly life cycle. Normalization against a housekeeping gene coding for ribosomal protein rp49 indicates that the levels of *HSPC300 *transcripts progressively increase during development (Figure 1a) and are particularly high in adult males and females. Transcript accumulation in ovaries is in line with high levels of *HSPC300 *RNA detected by *in situ *hybridization in embryos prior to onset of zygotic gene expression (data not shown).

A polyclonal antibody raised against the HSPC300 protein reveals a single band at the predicted molecular weight (8 kDa) in a western blot on *Drosophila *S2 cell extracts. Signal specificity was demonstrated first, upon transient *HSPC300 *overexpression, which induces significant enhancement of the 8 kDa band intensity (Figure 1b), and second, by loss of immunoreactivity in HSPC300 mutant extracts (see below). In embryos, HSPC300 primarily accumulates in the central nervous system (CNS), most prominent labeling localizing in axons along longitudinal tracts and commissures (Figure 1c). This expression pattern perfectly matches those previously reported for other components of the WAVE/SCAR complex [7,8]. Immunolabeling specificity was further confirmed by loss of immunoreactivity in *HSPC300 *mutants (see below).

### Generation of HSPC300 mutants

**Figure 2:**
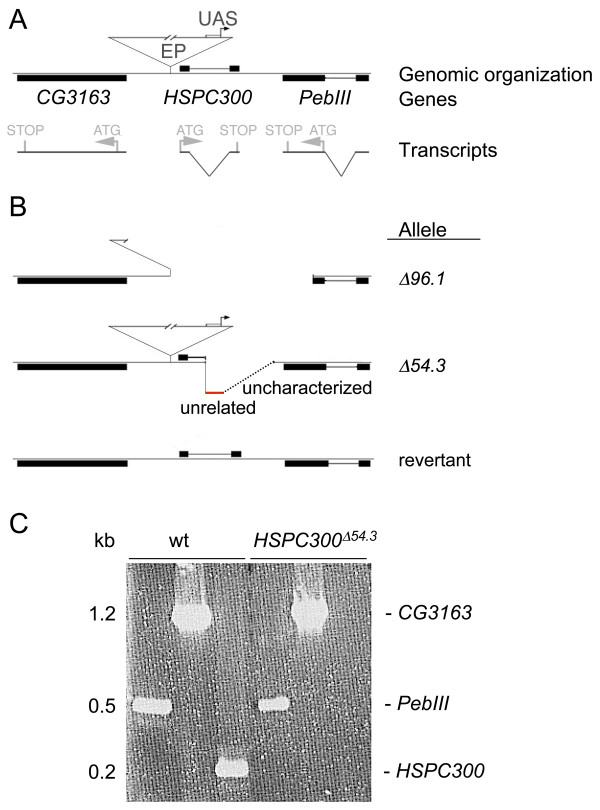
*HSPC300 *locus and mutants. **(a) **Exon/intron organization and resulting transcripts are shown for the genes *PebIII*, *CG3163 *and *HSPC300*. ATG and Stop (in grey) indicate open reading frames. EP indicates the P element insertion in the *EP(2R)0506 *line and UAS indicates the upstream activating sequence contained in the P element. **(b) **Molecular characterization of three excision lines. Line *HSPC300*^Δ*96.1*^(as well as non-depicted *HSPC300*^Δ*52.1*^*, HSPC300*^Δ*31.1 *^alleles) represent an excision event affecting the entire *HSPC300 *gene and part of the *PebIII *gene. Line *HSPC300*^Δ*54.3 *^displays intact adjacent genes (*PebIII *and *CG3163*) and P element. The red line represents integrated *HSPC300*-unrelated sequence, and the dotted line represents uncharacterized sequence. **(c) **RT-PCR on the wild-type (wt) and the *HSPC300*^Δ*54.3 *^excision line. The left three lanes show products obtained from wild-type third instar larvae; the right three lanes show product obtained from *HSPC300*^Δ*54.3 *^larvae. Note that *HSPC300 *transcripts are detected in the wild type (third lane from the left), but not in *HSPC300*^Δ*54.3 *^larvae (sixth lane from the left). In contrast, both *PebIII *(fourth lane from the left) and *CG3163 *(fifth lane from the left) transcripts are detected in the wild type and in mutant larvae.

To address the role of *HSPC300*, we generated loss of function mutants. The *EP(2R)0506 *line harbors a P element on the right arm of the second chromosome at position 60B4 and is 100% homozygous viable. The P element lies 75 bases upstream of the HSPC300 start codon (ATG), and in the right orientation to drive *HSPC300 *gene expression (Figure 2a). *HSPC300 *is flanked by two genes for which no mutant strain is reported, *CG3163*, located 263 bases upstream, and *PebIII*, located 497 bases downstream (Figure 2a).

We generated *HSPC300 *alleles by imprecise excision of the *EP(2R)0506 *P element and recovered 116 independent excision events. Among them, four lines are homozygous lethal at late pupal stage and fail to complement each other. These excision alleles were characterized by PCR and sequencing in order to identify the breakpoints. In three of them, the entire HSPC300 coding sequences are deleted, as well as part of the neighboring *PebIII *gene (Figure 2b and data not shown). The fourth lethal excision line, *HSPC300*^Δ*54.3*^, shows unaffected *PebIII *and *CG3163 *genes as well as the presence of an intact P element. In this line, the P element is unable to drive expression of *HSPC300 *as is the case with the original *EP(2R)0506 *line. To figure out the molecular lesions associated with this allele, we performed 3' and 5' inverse PCR. This confirmed the presence of intact junctions between the P element and surrounding genomic regions and revealed that the 3' end of the P element is flanked by a stretch of 208 bases of *HSPC300 *sequences followed by an insertion of unrelated sequences into the *HSPC300 *intron (see Figure 2b for schematic representation and Additional file 1 for molecular characterization of *HSPC300*^Δ*54.3*^). We characterized this allele further by RT-PCR and detected *PebIII *and *CG3163*, but no *HSPC300 *transcripts (Figure 2c), indicating that the modified *HSPC300 *transcript is either not transcribed or very rapidly degraded. Thus, *HSPC300*^Δ*54.3 *^represents a null allele that does not affect neighboring genes. This line was further used to characterize the mutant phenotypes and is referred to as *HSPC300 *unless otherwise indicated.

To provide formal evidence that excision line lethality is specifically due to loss of HSPC300, we generated *UAS-HSPC300 *transgenic animals and performed rescue experiments. Lethality observed in *HSPC300*^Δ*54.3 *^and *HSPC300*^Δ*96.1 *^lines is indeed rescued by *HSPC300 *re-expression using either *actin-Gal4 *or *elav-Gal4 *drivers (ubiquitous and pan-neuronal expression, respectively, data not shown). Altogether, these analyses demonstrate that *HSPC300 *is an essential gene required in the nervous system, its zygotic depletion inducing lethality prior to eclosion.

### HSPC300 is maternally contributed and is required for embryonic CNS axon morphology

**Figure 3:**
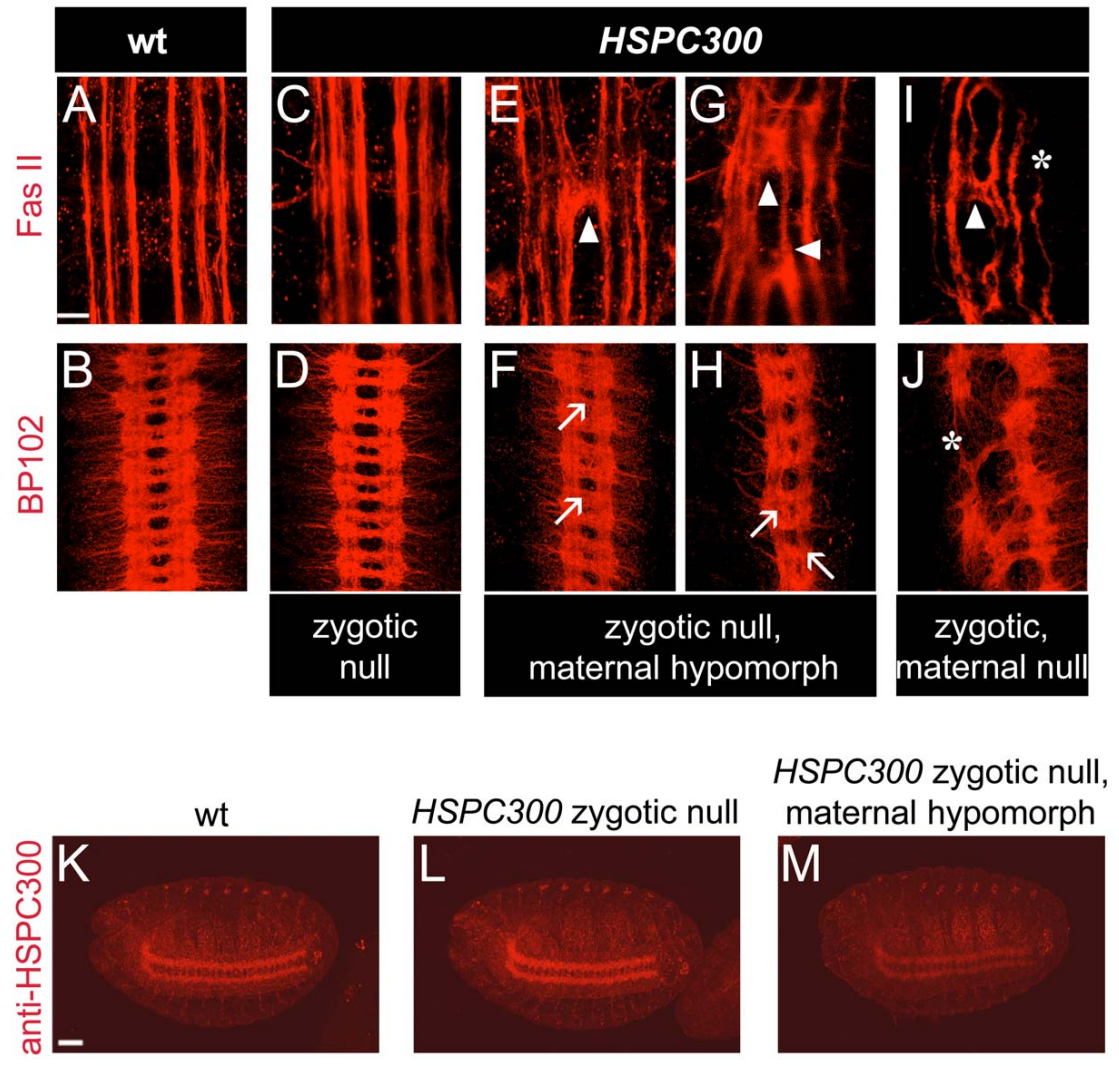
*HSPC300 *is maternally provided and is required in the morphology of embryonic CNS. **(a-j) **Embryos of indicated genotypes labeled with the axon-specific FasII and BP102 antibodies. All images show a portion of the ventral nerve cord (ventral views, anterior to the top) of stage 16/17 embryos. In wild-type embryos, anti-FasII reveals six longitudinal bundles (a), and BP102 two commissural bundles (b) in each segment. (c,d) *HSPC300 *embryos show CNS axon morphology that appears wild type. (e-h) Embryos that lack the HSPC300 zygotic component and are maternal hypomorphs show abnormal midline crossing (arrowhead) (e). In the most severe cases (g), axons ectopically cross the midline several times. (f,h) Commissures and longitudinal connectives are not properly formed (arrows). (i,j) Embryos that completely lack zygotic as well as maternal HSPC300 components show strongly disturbed CNS development, broken longitudinal connectives and commissures (asterisks). Scale bar: 20 μm. **(k-m) **Comparative analysis of HSPC300 expression in embryos of indicated genotypes; ventral views of stage 16 embryos, anterior to the left, anti-HSPC300 labeling. This experiment indicates that HSPC300 is maternally contributed and confirms the specificity of antibody. Scale bar: 50 μm.

The prominent axonal localization of HSPC300 and phenotypes previously reported for mutations in other components of the WAVE/SCAR complex [6-9] suggested a role for HSPC300 in axonogenesis. We therefore analyzed the integrity of the embryonic axonal network by immunolabeling with anti-FasII, which specifically recognizes six longitudinal fascicles of the CNS and motor axons, as well as with anti-BP102, which reveals connectives and commissures [32,33] (Figure 3). In contrast to the results obtained with *CYFIP*, *SCAR *and *Kette *mutants, complete loss of zygotic *HSPC300 *(*HSPC300*^Δ*54.3 *^and *HSPC300*^Δ*96.1*^) does not induce any prominent axon defect (Figure 3c,d). We speculated that maternal HSPC300 contribution compensates for loss of zygotic gene function, masking an essential requirement during embryogenesis. To test this hypothesis, we generated homozygous *HSPC300*^Δ*54.3 *^mutant clones within the germ line of heterozygous females. Deletion of both maternal and zygotic HSPC300 components leads to severe, though variable, nervous system defects ranging from broken and disorganized longitudinal connectives to remnants of axons or depletion of all CNS axon bundles (Figure 3i,j). Moreover, upon depletion of both maternal and zygotic HSPC300 components, lethality is shifted to embryonic stages and embryos appear overall disorganized. Identical observations were made using other *HSPC300 *alleles, such as *HSPC300*^Δ96.1 ^(data not shown).

The observed strong and pleiotropic defects did not allow us to score for specific neuronal requirement. We therefore aimed at reducing HSPC300 maternal dose without completely deleting it, using insertion line *EP(2R)0506*, referred to as *HSPC300*^*EP0506 *^from now onwards. This line represents a hypomorphic allele since *HSPC300*^*EP0506*^*/HSPC300*^Δ*54.3 *^embryos show 30% viability (versus 100% viability for *HSPC300*^*EP0506*^, and 0% viability for *HSPC300*^Δ*54.3*^). *HSPC300*^*EP0506*^*/HSPC300*^Δ*54.3 *^females were mated to heterozygous *HSPC300*^Δ*54.3 *^males. Amongst the progeny, we indeed noticed embryos with low maternal HSPC300 dose, reflected by reduced axonal HSPC300 immunolabeling in comparison with that observed in wild-type or zygotic null *HSPC300 *embryos under the same confocal microscope settings (Figure 3k–m). The resulting reduction in maternal HSPC300 dose produces embryos that overall show normal morphology (in contrast to those that are completely devoid of maternal HSPC300), allowing us to examine late CNS development. The embryonic phenotypes observed in this mutant condition, designated as maternal hypomorph, are similar, but of lower penetrance compared to those shown by other members of the WAVE/SCAR complex (midline crossing in 10% of embryos (n = 100) compared to 79% in *CYFIP *embryos (n = 150) [7]) (Figure 3e,g). Additional defects shared by mutants affecting the other subunits of the WAVE/SCAR complex [8] are disorganized fascicles, improper separation of commissures and broken longitudinal bundles (Figure 3e–h). Taken together, these data clearly implicate HSPC300 in axonogenesis, presumably through regulation of actin cytoskeleton remodeling by SCAR- and Arp2/3-dependent mechanisms. They moreover indicate that the HSPC300 maternal component is sufficient to sustain development in embryos that are devoid of zygotic HSPC300.

### HSPC300 regulates synaptic morphology at the NMJ

In addition to the embryonic axonal defects, mutations in other subunits of the WAVE/SCAR complex lead to synaptic defects that are characterized by size reduction of the NMJ and by supernumerary buds on pre-existing boutons [7,8,19]. The fact that *HSPC300 *as well as *CYFIP*, *SCAR *and *Kette *mutants show similar embryonic axonal defects prompted us to examine the structures of synaptic terminals in *HSPC300 *larvae.

**Figure 4:**
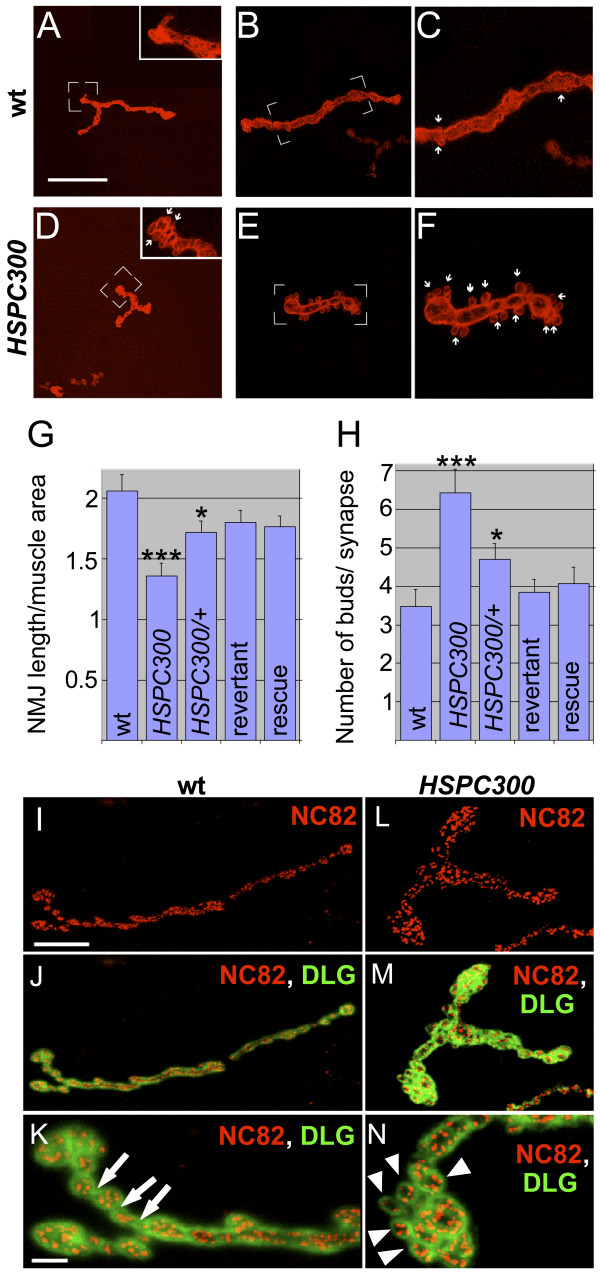
*HSPC300 *controls synaptogenesis at NMJs. **(a-f) **Anti-Disc Large immunolabeling of muscle 4 synaptic terminals of third instar larvae of the following genotypes: (a-c) wild type; (d-f)*HSPC300*. Compared to the wild type (WT), *HSPC300 *NMJs are shorter and display supernumerary buds. Insets in (a,d) show high magnifications of marked regions; (c,f) represent high magnifications of marked regions in (b,e), respectively. Arrows indicate buds. Scale bar: 20 μm (a,d); 14 μm (b,e); 8 μm (c,f). **(g,h) **Statistical evaluation of the NMJ phenotype of the following genotypes: wild type (WT); *HSPC300 *(*HSPC300*^Δ*54.3*^); *HSPC300 *heterozygous (*HSPC300*^Δ*54.3*^*/+*); Revertant, precise excision event (*HSPC300*^*86.1*^); and Rescue (*elav-Gal4; UAS-HSPC300, HSPC300*^Δ*54.3*^). (g) Length of synaptic terminals (μm) normalized over respective muscle surface area (μm^2^). (h) Number of buds per synapse. The sample size (number of muscle 4 junctions scored) was 25 per genotype. Error bars indicate SEM. Statistical significance (*p*) was calculated using ANOVA and *post hoc *analysis (see Materials and methods). Asterisks indicate *p*-value; *p < 0.05; ***p < 0.0001. **(i-n) **Wild type (i-k) and *HSPC300 *(l-n) NMJs labeled with anti-Disc Large (DLG; green) and active zone-specific marker NC82 antibody (red). Arrows indicate the region of low NC82 accumulation located between boutons, and arrowheads the supernumerary buds in the mutant synapse. Scale bar: 20 μm (i,j,l,m); 4 μm (k,n).

NMJs were labeled using anti-Disc Large antibody, a preferred marker to reveal NMJ morphology [7,8,34]. NMJs of *HSPC300 *larvae are severely reduced in length and display supernumerary buds (Figure 4d–h) compared to those of wild-type animals, those of rescued animals or those observed in a precise excision line (Figure 4a–c,g,h). The latter represents a revertant as it displays the same genetic background as that of imprecise excision lines (Figure 4g,h).

To quantitatively assess synaptic *HSPC300 *phenotypes, we measured total length of synaptic terminals using a computer-assisted program [7] and counted buds per synaptic terminal. We focused our analysis on muscle 4 in abdominal segments A2-A4, innervated by the inter-segmental nerve (ISN). Since NMJ growth varies in proportion to muscle size [35], we normalized the total length of each synapse with its corresponding muscle surface area. Normalized synapse length of *HSPC300 *larvae corresponds to 66% of the wild-type NMJ length, a statistically highly significant difference (1.36 × 10^-3 ^± 0.11 μm^-1 ^versus 2.06 × 10^-3 ^± 0.137 μm^-1 ^(mean ± standard error of the mean (SEM)); *p *< 0.001). Likewise, there is 88% increase in the bud number compared to wild type (6.42 ± 0.60 versus 3.51 ± 0.39 (mean ± SEM); *p *< 0.001) (Figure 4g,h). Strong labeling with active zone-specific antibody NC82, which recognizes the Bruchpilot protein [35,36], indicates that *HSPC300 *synapses are functional (Figure 4i–n). Interestingly, compared to wild-type NMJs, in which NC82 labeling preferentially localizes at boutons, mutant NMJs show uniform labeling. This is in line with the finding that the characteristic bead string structure of NMJs is not preserved in *HSPC300 *larvae, in which boutons cannot be easily identified. This phenotype is shared by mutations affecting the other subunits of the WAVE/SCAR complex [7,8,37] and may be related to the supernumerary budding phenotype.

Finally, NMJ phenotypes are rescued by *elav*-*Gal4*-driven *HSPC300 *expression but not by postsynaptic *HSPC300 *expression induced by the muscle-specific *mhc-Gal4 *driver (Figure 4g,h, and data not shown). Taken together, these results reveal that HSPC300 is required by motoneurons for synaptic morphogenesis.

### HSPC300 in the context of the WAVE/SCAR complex

**Figure 5:**
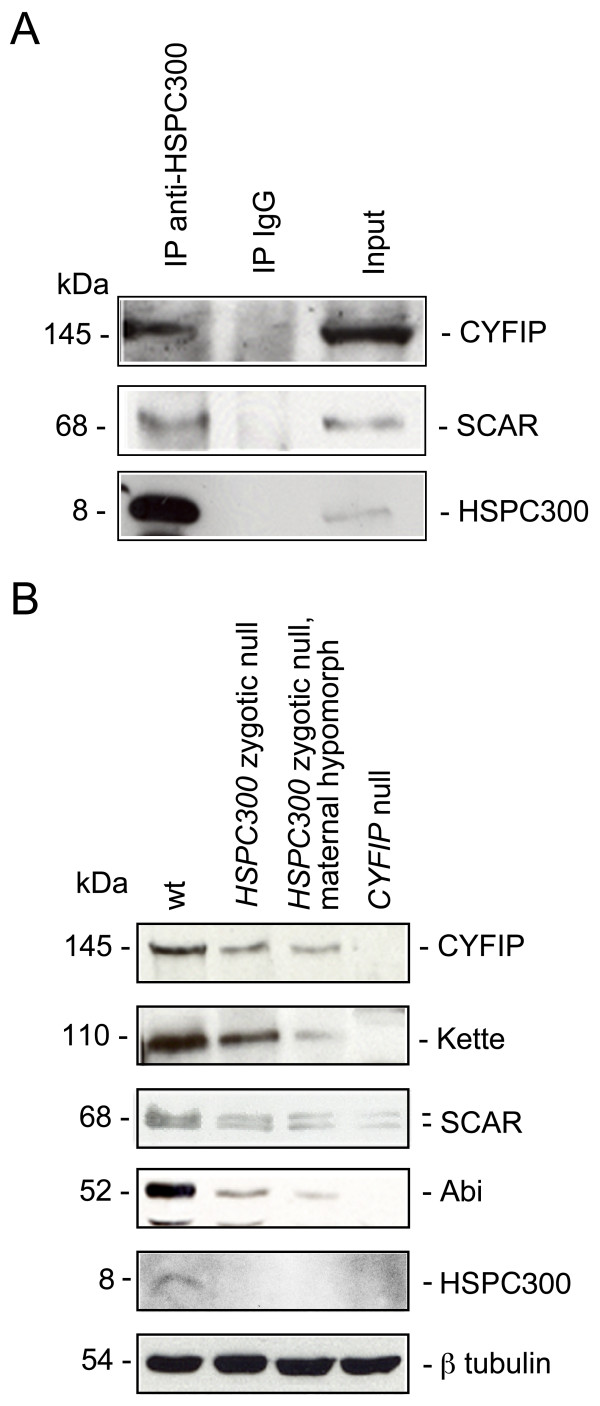
Protein levels of WAVE/SCAR complex subunits in different mutant contexts. **(a) **Immunoprecipitation experiments in *Drosophila *S2 cytoplasmic cell extracts using the anti-HSPC300 antibody. From left to right: anti-HSPC300 immunoprecipitation, IgG immunoprecipitation, input (cytoplasmic extract). Proteins are indicated to the right, corresponding molecular weights to the left. **(b) **Quantitative analysis of CYFIP, Kette, SCAR, Abi and HSPC300 protein levels by western blot on third instar larval extracts of the following genotypes: wild type (WT); *HSPC300 *zygotic null; *HSPC300 *zygotic null and maternal hypomorph; *CYFIP *zygotic null. Proteins are indicated to the right, corresponding molecular weights to the left. β-tubulin represents a loading control.

The phenotypic similarity of axonal and synaptic architecture of *HSPC300*, *CYFIP*, *Kette *and *SCAR *mutants strongly suggests synergy amongst the wild-type molecules and indicates that HSPC300 represents a functional subunit of the *Drosophila *WAVE/SCAR complex *in vivo*. To provide evidence for the physical association of HSPC300 and the other WAVE/SCAR complex subunits, we performed co-immunoprecipitation experiments in *Drosophila *S2 cells. Both CYFIP and SCAR proteins were found to co-immunoprecipitate with HSPC300 (Figure 5a), as revealed by using anti-CYFIP (band at 145 kDa) and anti-SCAR (68 kDa) antibodies [7,9]. Control experiments performed in parallel with unrelated rabbit IgG did not co-immunoprecipitate either of these proteins, thus confirming the specificity of the observed interactions. Thus, *Drosophila *HSPC300 appears to represent, like its mammalian counterpart, a subunit of the WAVE/SCAR complex.

We and others have previously demonstrated that CYFIP, Kette and SCAR are essential for the integrity and the stability of the WAVE/SCAR complex *in vivo*. If any of them is missing, the other subunits fail to be detected [8,21-25], most likely due to proteasome-mediated degradation [23]. In line with the observation that zygotic *HSPC300 *null embryos show neither significant reduction of HSPC300 labeling (Figure 3l) nor axonal defects (Figure 3c,d), other WAVE/SCAR complex proteins (SCAR and CYFIP) accumulate at wild-type levels in these embryos (Additional file 2). In order to determine the stabilizing potential of HSPC300, we therefore assessed the levels of CYFIP and SCAR later in development, in third instar *HSPC300 *mutant larvae, a developmental stage in which maternal contribution has faded. Indeed, we noticed a considerable decrease in CYFIP, Kette, SCAR and Abi protein levels compared to those observed in wild-type larvae. Qualitatively similar results were obtained in zygotic null larvae and in larvae that completely lack the zygotic component and also are maternal hypomorphs, even though the latter genotype shows a stronger defect (Figure 5b). Interestingly, there appears to be yet stronger reduction of these proteins in the extracts prepared from *CYFIP *zygotic null animals (Figure 5b).

To provide formal evidence that *HSPC300*-induced phenotypes depend on its functional relation with the WAVE/SCAR complex, we performed genetic interaction experiments at the NMJ. We took advantage of our previous observation that single mutations in the subunits of the WAVE/SCAR complex show reduced synapse length in a dose-dependent manner. Heterozygous mutants indeed already show significantly reduced synaptic length [8] (Figure 4g). Interestingly, while double heterozygous *HSPC300*, *CYFIP *NMJs are similar to those from larvae that are heterozygous for either gene, imbalance between HSPC and CYFIP content using a sensitized background results in a marked further decrease of synaptic terminal length: the strong *CYFIP *transheterozygous allelic combination *CYFIP*^Δ*85.1*^*/CYFIP*^*EP3267 *^(that is, null over hypomorph), in an otherwise heterozygous *HSPC300 *background, leads to a significant reduction in the synapse length (Table 1). This result indicates that *HSPC300 *and *CYFIP *synergistically affect synapse growth and provides a first assessment of the molecular pathway requiring HSPC300.

**Table 1:** Genetic interactions at NMJs

Genotype	Mean Synapse length ± SEM (μm)	*P*-values
Wild type	87.46 ± 2.28	
*HSPC300*^Δ*54.3*^/+	78.48 ± 3.88	*P*^*Wild type*^< 0.05
*CYFIP*^Δ*85.1*^/+	78.83 ± 2.92	P^Wild type^< 0.05
*CYFIP*^Δ*85.1*^/*CYFIP*^*EP3267*^	68.18 ± 3.18	
*HSPC300*^Δ*54.3*^/+; *CYFIP*^Δ*85.1*^/+	80.47 ± 2.52	
*HSPC300*^Δ*54.3*^/+;	62.00 ± 2.69	*P*^*CYFIP*/*CYFIPEP3267*^~0.142
*CYFIP*^Δ*85.1*^/*CYFIP*^*EP3267*^		

The sample size per genotype in all cases was 25. *P*-values versus indicated genotypes (superscript) were determined by Student's *t*-test.

**Figure 6:**
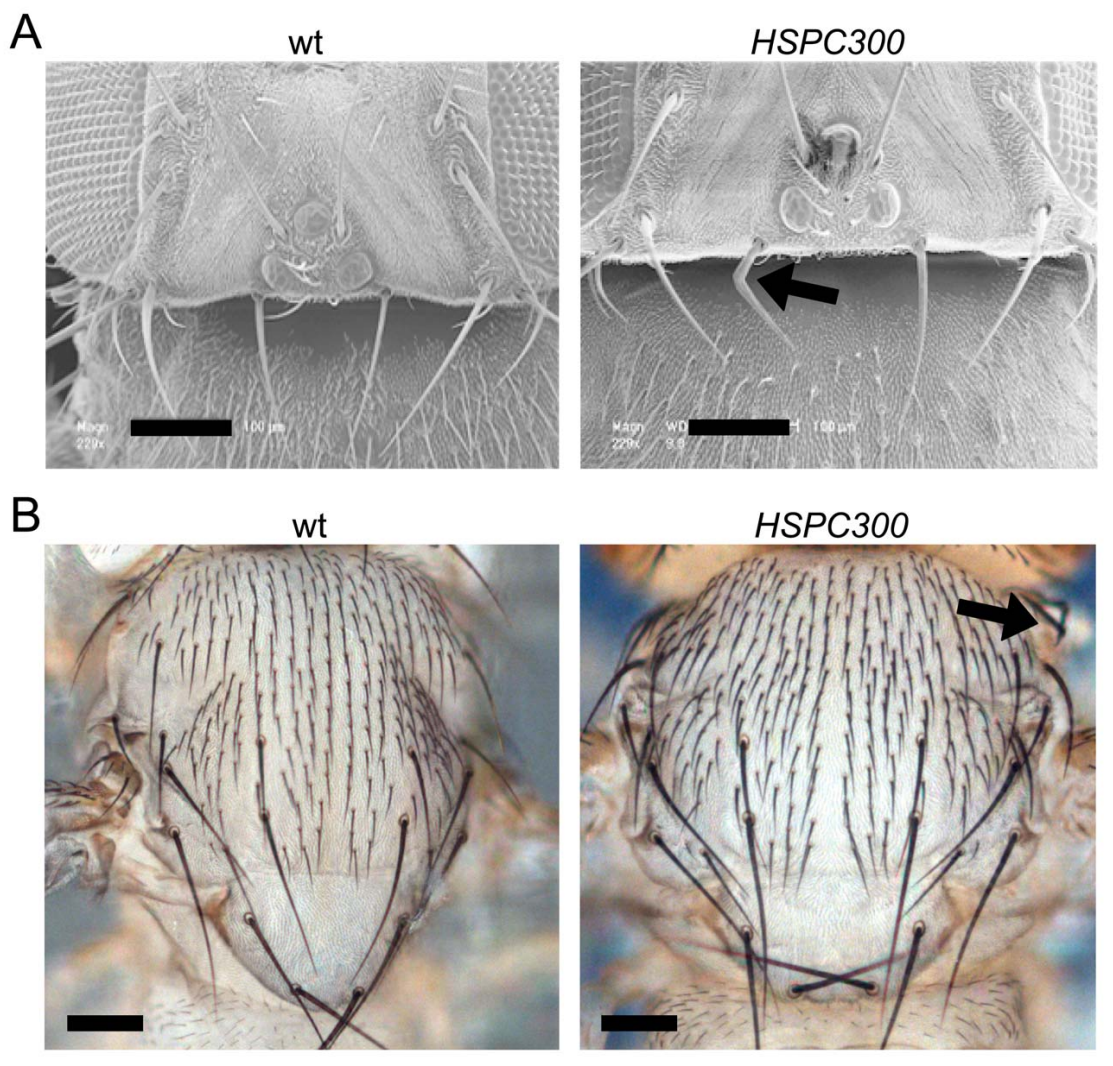
*HSPC300 *Bristle phenotype. **(a) **Scanning electron microscope images of the dorso-posterior portion of the *Drosophila *head and **(b) **light microscope images of thoracic bristles. On the left are wild-type (wt), and on the right *HSPC300 *pharate adults. Occasionally, bristles in *HSPC300 *pharate adults show typical bent morphology (arrows). Note that *HSPC300 *pharate adults display normal specification of sensory bristles on the head, notum and scutellum. Scale bars: 100 μm.

The above data clearly demonstrate that HSPC300 animals share nervous system defects with *SCAR*, *CYFIP *and *Kette *mutants. Flies mutant for subunits of the WAVE/SCAR complex are also characterized by misshaped macrochaetae [5,19,20], a phenotype due to defects in F-actin. The phenotype of bent bristles is also observed in *HSPC300 *pharate adults (Figure 6, arrows). However, specification of sensory organ precursors (SOPs) is not affected and the number of bristles on the head and thorax are normal (Figure 6), as in mutant *SCAR *and *CYFIP *conditions [9] (our unpublished data). This result is particularly interesting in view of the observation that HSPC300 also directly interacts with Abi [29], which in turn interacts with and activates WASP [5,30]. The Abi-WASP biochemical interaction is further sustained by the common phenotype of *WASP *and knockdown *Abi *mutant flies, which lack macrochaete on the thorax, due to SOP cell fate defects [5,20]. On the basis of the phenotypical similarity, we conclude that loss of HSPC300 affects signal transduction through SCAR, not WASP.

## Discussion

The evolutionarily conserved WAVE/SCAR complex is known to regulate the actin nucleating Arp2/3 complex in a Rac-dependent manner [1,2,7,8,23,27,38]. While the functions of most components of this complex have been extensively explored, the role of the small protein HSPC300, although one of the complex core subunits, is less understood. Here, we present the first functional analysis of HSPC300 in an animal model and demonstrate the importance of this protein in nervous system development and connectivity.

### HSPC300 functions within the Rac1-WAVE/SCAR complex-Arp2/3 pathway

The SCAR, CYFIP, Kette and Abi subunits of the WAVE/SCAR complex have been shown to impact on various processes and structures that depend on actin cytoskeletal remodeling. These include *Dictyostelium *motility [21], development of plant trichomes [27], as well as egg chamber structure and nuclear positioning in the blastoderm of *Drosophila *[9]. Crucial for the versatile functions of the complex is its structural integrity. Loss or mutations in any subunit (CYFIP, Kette or SCAR) leads to proteasome-mediated degradation of the others and, as a consequence, identical mutant phenotypes [8,21,23]. Surprisingly, *HSPC300 *knockdown in *Drosophila *S2 cells has been reported to produce much milder cytoskeletal phenotypes than those produced by targeting other components of the WAVE/SCAR complex [23]. In addition, in *in vitro *experiments, lack of HSPC300 affects neither complex assembly [29] nor the complex's ability to activate Arp2/3 *in vitro *[22], two features that characterize other complex subunits. These data raise questions as to the real role of *HSPC300 *in the context of the WAVE/SCAR complex.

Presented data demonstrate that *Drosophila *HSPC300 constitutes a subunit of the WAVE/SCAR complex *in vitro *and *in vivo*. Like the other subunits of the complex, *HSPC300 *is highly expressed in the developing fly nervous system and is crucially required for axonogenesis and neuromuscular synapse morphogenesis. Moreover, *HSPC300 *loss of function conditions are marked by a decrease in all members of the WAVE/SCAR complex, a result that is in keeping with a model in which each subunit, including HSPC300, significantly contributes towards the stability of the complex, notably *in vivo*. By using *HSPC300 *allele combinations that lead to different amounts of HSPC300 protein, we have revealed that a sharp threshold exists and that the maternal component is sufficient to ensure normal embryonic development and viability. The stringent genetic conditions we generated (loss of zygotic in addition to partial or complete loss of maternal HSPC300) also allowed us to reveal that strong loss of HSPC300 protein is necessary to cause dramatic consequences comparable to those observed in mutants affecting other complex components, thereby suggesting that the previously obtained mild phenotypes (RNAi-mediated knockdown in cells [23]) merely result from the limitation of the utilized technique. Thus, highly similar requirements for HSPC300 and the WAVE/SCAR complex components control cell morphology in CNS neurons and at NMJs.

The genetic interaction observed upon disrupting the balance between CYFIP and HSPC300 levels shows a positive/synergistic role for HSPC300 in the Rac1-WAVE/SCAR complex pathway to control synapse length, thereby providing first genetic evidence for HSPC300 functioning in this pathway. Taken together, the phenotypes and genetic interactions in the fly nervous system, as well as the phenotypes previously described in *Arabidopsis *[26,27], provide strong evidence for HSPC300 being an evolutionarily important integral part of the Rac1-WAVE/SCAR-Arp2/3 pathway.

Interestingly, it has been shown that the control of cotyledon cell size requires *Arabidopsis *Brick1 but not WAVE/SCAR-Arp2/3 [27], suggestive of an additional function that does not depend on the WAVE/SCAR complex. These data are in line with the existence of a significant fraction of free and soluble vertebrate HSPC300 and with our finding that phenotype and genetic interactions can only be revealed upon strong HSPC300 depletion and imbalance, respectively. Whether HSPC300, similar to CYFIP, Kette and Abi, works on additional pathways that are independent of the WAVE/SCAR complex remains to be elucidated. Since HSPC300 is the most conserved subunit of the WAVE/SCAR complex not only in the animal kingdom and *Dictyostelium*, but also in plants, and since no HSPC300 paralogous gene exists in flies, we expect our data to be of predictive value for HSPC300 indispensability with respect to the function of its associated complex in other organisms. We further expect the generated mutant animals to facilitate identification of novel HSPC300-dependent pathways.

## Conclusion

We present the first evidence that the small protein HSPC300 is an indispensable component of the WAVE/SCAR complex and plays an important role in nervous system development in *Drosophila*. Moreover, accumulating data suggest that signaling of the small Rho GTPase Rac1 through the WAVE/SCAR complex is implicated not only in structural connectivity in fly [5-9] (this study) and mouse [3,4] brain, but is also involved in higher cognitive functions and human disease when mutated. Mutations in at least a dozen genes associated with mental retardation in humans directly regulate/mediate Rho GTPase function or may be connected to their associated signaling pathways (reviewed in [39-42]). This notably includes two genes the products of which directly associate with the WAVE/SCAR complex: FMRP [7,8,13] and a GTPase activating protein termed MEGAP/WRP/srGAP3 [43,44]. These findings emphasize the dominant role of this complex not only in architecture but also in higher functions of the nervous system. Moreover, they place the WAVE/SCAR complex in a central position to genes that are highly relevant to cognitive functions. Based on present data, we propose that *HSPC300 *is a new promising candidate gene for genetic causes underlying impaired cognition. Whereas final evidence for the role of *HSPC300 *in mammal cognition awaits the characterization of *HSPC300 *mouse knockout phenotypes, a *WAVE *knockout mouse does indeed show learning and memory defects [43,44] and altered synaptic plasticity [4].

## Materials and methods

### Genetics

The wild-type strain used was *Sevelen*. Line *EP(2R)0506 *was provided by the Szeged Stock Center (Szeged, Hungary), and *elav*-*Gal4 (C155) *by the Bloomington Stock Center (Bloomington, Indiana, USA). *HSPC300 *excision mutants were obtained upon transposon mobilization in the *EP(2R)0506 *line after isogenization. Transgenic lines were obtained using standard protocols. *UAS-HSPC300 *(line 4b.1) was recombined onto the *HSPC300*^Δ*54.3 *^carrying chromosome. *FRT42B *was recombined separately with *HSPC300*^Δ*54.3 *^and *HSPC300*^Δ*96*^. Germ line clones were induced in *hs-FLP; FRT42B Ovo*^*D*^/*FRT42B*, *HSPC300*^Δ*54.3 *^flies as described [45]. Other utilized strains were *CYFIP*^Δ*85.1*^, *Kette*^*03335 *^and *SCAR*^Δ*37 *^[8].

### Molecular techniques

#### DNA constructs

*HSPC300 *coding sequence was amplified by RT-PCR on total RNA extract from *Drosophila *S2 cells. Utilized primers were GGG GAA TTC AAA GAT GAG TGG GGC TCA CAG and GGG CTC GAG CGT TTA CGT TAA TGT TTC ACC CTG. After *Eco*RI, *Xho*I digestion, the fragment was cloned into pGEM-T and pUAST vectors.

#### Quantitative mRNA analysis

Total RNA was recovered from animals of the indicated developmental stage or tissue and subjected to cDNA synthesis using primers mentioned above. Real time PCR was performed according to standard protocols with a Light Cycler (Roche, Basel, Switzerland) using primers AAA GCA GAT CCA CCA GGA CT and CGC TCC AGG ATC GTT AGT TT. Primers for housekeeping gene *rp49 *were GCG CAC CAA GCA CTT CAT C and GAC GCA CTC TGT TGT CGA TAC C. All samples were analyzed in replicates of four.

#### 5' and 3' Inverse PCR

Total genomic DNA of *HSPC300*^Δ*54.3 *^flies was extracted by standard methods, digested with *Msp*I and *Eco*RI, followed by self ligations in separate reactions. *Msp*I severed within the P element and flanking 3' sequence, resulting in rescue of the 3' end. *Eco*RI severed within the P element and flanking 5' sequence, resulting in rescue of the 5' end. The rescued ends were amplified by standard primer combination of GTA ACG CTA ATC ACT CCG AAC AGG TCA CA and CAA TCA TAT CGC TGT CTC ACT CA. Amplified products were subsequently sequence verified by ACA CAA CCT TTC CTC TCA ACA A for the 5' end and GAC ACT CAG AAT ACT ATT C for the 3' end.

#### Immunolabeling on embryos and larvae

Immunolabeling on embryos was performed according to standard procedures. Larvae open-book preparations and immunolabeling are described in Bellen and Budnik [46]. Antibodies utilized were: polyclonal HSPC300 antibody (1:1,000), anti-Fas II ID4 (1:50) and anti-Disc large (1:20) (Developmental Studies Hybridoma Bank, Iowa City, USA), anti-GFP (1:50; Santa Cruz Biotechnologies (Santa Cruz, California, USA), anti-SCAR [9] (1:100) and anti-CYFIP [8] (1:100).

#### Immunoprecipitations

S2 cells were cultured in Schneider cell medium (Gibco BRL/Invitrogen, Karlsruhe, Germany) + 10% fetal calf serum. Cytoplasmic extracts were prepared by lysing S2 cells in buffer (200 mM NaCl, 20 mM Tris-HCl pH 7.5, 5 mM MgCl_2_, 0.4% Triton X-100, protease inhibitor cocktail (PIC)), kept on ice for 10 minutes. The 2,000 g supernatant was incubated for 6 h with 5 μg of either anti-HSPC300 or rabbit IgG and protein A Sepharose. Beads were extensively washed in lysis buffer, directly boiled in SDS-PAGE loading buffer and subjected to SDS-PAGE analysis.

#### Embryonic and larval extracts

Animals of the correct genotype were collected on the basis of labeled balancer chromosomes. Embryonic extracts were prepared from overnight (18 h) cages. Embryos and wandering third instar larvae were mashed using pestles in 150 mM NaCl, 20 mM Tris-HCl pH 7.5, 1 mM EDTA, 0.1 mM MgCl_2_, 1% Triton X-100, PIC, followed by an incubation on ice for 10 minutes. The supernatant of a 12,000 g centrifugation was briefly sonicated, and the amount of total protein was determined by Bradford assay.

#### Western blot analysis

Proteins were separated in 7% polyacrylamide gels. For detection of HSPC300, proteins were resolved on precast 4–12% Bis-Tris polyacrylamide gels (Invitrogen, Karlsruhe, Germany) in NuPAGE-MES buffer. SDS-PAGE and blotting were performed according to standard procedures. Primary antibodies used in western blot analysis were anti-HSPC300 (1:3,000), anti-CYFIP #1719 (1:100), anti-SCAR (1:2,000), anti-Kette #2081 [8] (1:2,000) and anti-β-tubulin (1:4,000) (Chemicon, Hampshire, UK).

#### Microscopy and statistics

The confocal microscope was a Leica TCS-SP1. Confocal images were assembled using in-house developed software. Synapse images for quantification were obtained using a Zeiss Axiophot 2 microscope. Scanning electron microscopy was performed with a Philips -XL20 microscope. Twenty-five NMJs (muscle 4, segments A2-A4) were scored per genotype. All pictures of synapses were taken at 40x and their corresponding muscle area pictures were taken at 10x and subjected to an in-house developed software [7,8]. Statistical significance was calculated using ANOVA and the Newman-Keuls method for *post hoc *pair-wise analyses.

## Competing interests

The author(s) declare that they have no competing interests.

## Authors' contributions

AQ generated HSPC300 mutant flies and drafted a version of the manuscript. AQ and BS carried out the phenotypic analysis. PC and AS participated in coordinating the study, AS also participated in designing it. AQ, BS, AS, and Angela G prepared the figures. Angela G conceived the study and wrote the final version of the manuscript, together with AS. Alexis G produced novel tools required for the study (anti-HSPC300 and anti-SCAR antibodies). All authors have read and approved the final version of the manuscript.

## Supplementary Material

Additional file 1 Molecular characterization of excision line *HSPC300*^Δ*54.3*^. Sequence obtained upon 5' and 3' inverse PCR (compared with original line *EP(2R)0506*) showing the presence of intact junctions between the P element and surrounding genomic sequences. Note that, following 208 bases of the *HSPC300 *sequence, an unrelated sequence of at least 74 bases is present. Click here for file

Additional file 2 Normal levels and localization of other WAVE/SCAR complex proteins (CYFIP, SCAR) in HSPC300 zygotic null embryos. **(a-f) **Wild-type (WT), HSPC300 zygotic null and, as control, CYFIP zygotic null embryos labeled with anti-CYFIP (a-c) or anti-SCAR (d-f) antibodies. **(g) **Anti-CYFIP and anti-SCAR immunoblot analysis of WT and HSPC300 zygotic null mutant extracts. Note that in contrast to genetic conditions in which maternal and zygotic HSPC300 doses have been depleted, there is no appreciable difference in CYFIP and SCAR levels and distribution in HSPC300 zygotic null embryos. Maternally provided HSPC300 protein is thus sufficient to stabilize other WAVE/SCAR complex proteins during embryonic development. In contrast, despite maternal contribution, loss of zygotic CYFIP destabilizes SCAR. Scale bar: 75 μm. Click here for file
