# Supplementary material for: HSPC300 and its role in neuronal connectivity
Source: Neural Dev. 2007 Sep 25;2:18. doi: 10.1186/1749-8104-2-18 (PMC2098765; doi:10.1186/1749-8104-2-18)
Supplement: Additional file 1 — Molecular characterization of excision line HSPC300Δ54.3. Sequence obtained upon 5' and 3' inverse PCR (compared with original line EP(2R)0506) showing the presence of intact junctions between the P element and surrounding genomic sequences. Note that, following 208 bases of the HSPC300 sequence, an unrelated sequence of at least 74 bases is present. [file 1749-8104-2-18-S1.doc]

**Additional File 1**

**
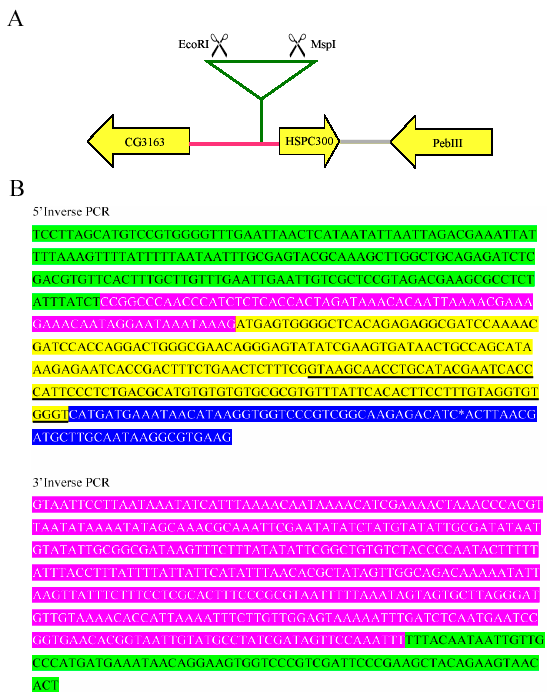
**

**Additional File 1:** Molecular characterization of excision line *HSPC300^Δ54.3^*.

(A) Schematic representation indicating P element insertion in *HSPC300^0506^* line and gene orientations. (B) Sequence data obtained upon 5' (top) and 3' (bottom) Inverse PCR on *HSPC300^Δ54.3^*, revealing intact P element junctions.

Color code: Green represents P element sequence, pink represents genomic sequences between P element and adjacent genes, yellow represents annotated Genes (in A) or *HSPC300* gene (in B). Blue (in B) indicates the *HSPC300*-unrelated integrated sequence. Underlined yellow sequence (in B) indicates *HSPC300* intron.
