# Supplementary figures and images for: HSPC300 and its role in neuronal connectivity
Source: Neural Dev. 2007 Sep 25;2:18. doi: 10.1186/1749-8104-2-18 (PMC2098765; doi:10.1186/1749-8104-2-18)

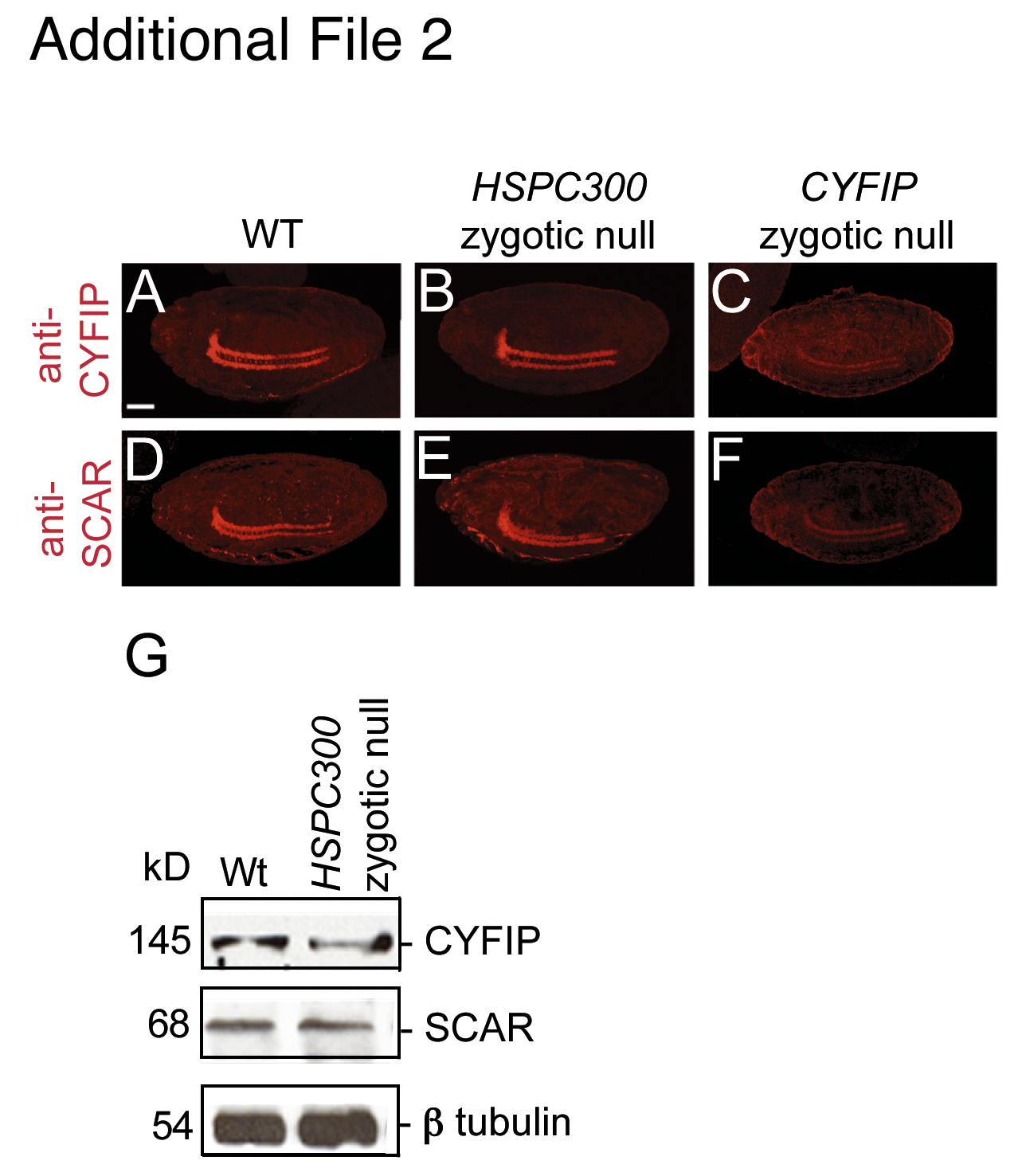

Supplement: Additional file 2 — Normal levels and localization of other WAVE/SCAR complex proteins (CYFIP, SCAR) in HSPC300 zygotic null embryos. (a-f) Wild-type (WT), HSPC300 zygotic null and, as control, CYFIP zygotic null embryos labeled with anti-CYFIP (a-c) or anti-SCAR (d-f) antibodies. (g) Anti-CYFIP and anti-SCAR immunoblot analysis of WT and HSPC300 zygotic null mutant extracts. Note that in contrast to genetic conditions in which maternal and zygotic HSPC300 doses have been depleted, there is no appreciable difference in CYFIP and SCAR levels and distribution in HSPC300 zygotic null embryos. Maternally provided HSPC300 protein is thus sufficient to stabilize other WAVE/SCAR complex proteins during embryonic development. In contrast, despite maternal contribution, loss of zygotic CYFIP destabilizes SCAR. Scale bar: 75 μm. [file 1749-8104-2-18-S2.png]
